# Cellular Mechanotransduction: From Tension to Function

**DOI:** 10.3389/fphys.2018.00824

**Published:** 2018-07-05

**Authors:** Fabiana Martino, Ana R. Perestrelo, Vladimír Vinarský, Stefania Pagliari, Giancarlo Forte

**Affiliations:** ^1^Center for Translational Medicine, International Clinical Research Center, St. Anne’s University Hospital, Brno, Czechia; ^2^Department of Biology, Faculty of Medicine, Masaryk University, Brno, Czechia; ^3^Competence Center for Mechanobiology in Regenerative Medicine, INTERREG ATCZ133, Brno, Czechia; ^4^Department of Biomaterials Science, Institute of Dentistry, University of Turku, Turku, Finland

**Keywords:** mechanotransduction, nucleoskeleton, focal adhesion, mechanobiology, mechanosensor

## Abstract

Living cells are constantly exposed to mechanical stimuli arising from the surrounding extracellular matrix (ECM) or from neighboring cells. The intracellular molecular processes through which such physical cues are transformed into a biological response are collectively dubbed as mechanotransduction and are of fundamental importance to help the cell timely adapt to the continuous dynamic modifications of the microenvironment. Local changes in ECM composition and mechanics are driven by a feed forward interplay between the cell and the matrix itself, with the first depositing ECM proteins that in turn will impact on the surrounding cells. As such, these changes occur regularly during tissue development and are a hallmark of the pathologies of aging. Only lately, though, the importance of mechanical cues in controlling cell function (e.g., proliferation, differentiation, migration) has been acknowledged. Here we provide a critical review of the recent insights into the molecular basis of cellular mechanotransduction, by analyzing how mechanical stimuli get transformed into a given biological response through the activation of a peculiar genetic program. Specifically, by recapitulating the processes involved in the interpretation of ECM remodeling by Focal Adhesions at cell-matrix interphase, we revise the role of cytoskeleton tension as the second messenger of the mechanotransduction process and the action of mechano-responsive shuttling proteins converging on stage and cell-specific transcription factors. Finally, we give few paradigmatic examples highlighting the emerging role of malfunctions in cell mechanosensing apparatus in the onset and progression of pathologies.

## Introduction

The correct regulation of cell function *in vivo* requires the integration of numerous biological and mechanical signals arising from the surrounding cells and the extracellular matrix (ECM).

The nanostructure and the composition of the ECM is strictly controlled in a tissue-specific fashion during development and in adulthood in order to favor cell and organ function (Smith et al., 2017). Changes in ECM composition and mechanics are encountered during the progression of all degenerative diseases as the result of aging or as a compensatory attempt of the tissue to preserve its function (Kim et al., 2000; Parker et al., 2014; Klaas et al., 2016). Changes in ECM compliance are now considered of prognostic value for solid tumors (Calvo et al., 2013; Hayashi and Iwata, 2015; Reid et al., 2017).

**Glossary:** 

Extracellular Matrix (ECM)	Network and reservoir of extracellular and signaling molecules which are secreted locally to ensure cell and tissue cohesion
Focal adhesions (FAs)	Integrin-based cell-matrix physical contacts that transduce and integrate mechanical and biochemical cues from the environment through the recruitment of intracellular multiprotein assemblies connected to actin cytoskeleton
Mechanosensing	The ability of a cell to sense mechanical cues of its micro-environment, including not only all components of force, stress and strain but also substrate rigidity, topography and adhesiveness.
Mechanotransduction	Molecular process transforming a physical stimulus in a biological response
Mechanotransducers	Individual or protein complexes that produce or enable a chemical signal in response to a mechanical stimulus
Mechanical instability	Is the result of substrate mechanical oscillations at cell-ECM interface, that induces internal cellular and molecular rearrangements, in order to recover to an equilibrium state
Tension	Pulling force transmitted axially by means of an object
Tensional homeostasis	A basal equilibrium stress state in which cells counteract external force application by moving toward a previous force setpoint that had been established before external force application
Stiffness	Resistance of an elastic body to deflection or deformation by an applied force
Stress fibers (SFs)	Bundles of F-actin and myosin II held together by cross-linking proteins ensuring the cytoskeletal contractility

The best example of how pathology-driven changes in ECM mechanics and architecture impact on tissue and organ function is cardiac remodeling. Following a cardiovascular event (ischemic insult, long term exposure to pressure or volume overload), cardiac matrix is degraded and substituted by a scar, having a different nanostructure and compliance (Spinale, 2007). The structural changes occurring within the myocardial ECM affect cardiomyocyte function (Engler et al., 2008), thus compromising the overall structure and function of the whole myocardium.

Similar effects of altered ECM mechanics on the function of different cell types have been recently demonstrated (Engler et al., 2006; Natarajan et al., 2015; Zarkoob et al., 2015).

The concept that cells can interpret and respond to mechanical cues is not exactly new to the scientific community. Nonetheless, only lately, the elucidation of the molecular mechanisms by which the cell perceives and transforms the mechanics of the ECM has become the subject of intense investigation and a number of intracellular molecules has been identified that can react to mechanical stimulation and - in turn – modify cell function.

So far, the definition of cellular mechanosensor has been applied to a number of molecules – mainly proteins – displaying a status change in response to mechanical stimulation. The nature and the degree of the change imposed by mechanical cues can vary significantly and post-translational modifications (Sawada et al., 2006; Dong et al., 2009; Hayakawa et al., 2011; Swift et al., 2013; Guilluy et al., 2014; Qin et al., 2015; Sathe et al., 2016; Lachowski et al., 2018), intracellular shuttling (Gumbiner, 1995; Gottardi et al., 1996; Huber et al., 1996; Lewis et al., 1996; Orsulic and Peifer, 1996; Nix and Beckerle, 1997; Dupont et al., 2011), protein unfolding (del Rio et al., 2009), the creation of novel interactions (Humphries et al., 2007) are considered as positive signatures of mechanical responsiveness. All these responses can be found during the transmission of mechanical signals from the ECM to the nucleus, a molecular process collectively known as mechanotransduction.

Cells perceive mechanical stimuli through diverse mechanosensitive molecules at the cell membrane including integrins, stretch-activated ion channels, G protein coupled-receptors, growth factor receptors, activating different mechanotransduction pathways (Martinac, 2014; Luis Alonso and Goldmann, 2016).

In the present review, we focus on the cellular mechanical response through ECM-integrin-cytoskeleton-nucleus axis and critically discuss the molecular basis of focal adhesion cell mechanosensing. We highlight how different intracellular molecules respond to mechanical loading and transfer the information from the very site of cell-ECM interaction – the membrane – to the nucleus, where the mechanosensitive genes are eventually activated.

## Focal Adhesions: the Main Hub for Cell-Matrix Interaction

The primary site of force transmission to the cell is the cellular membrane, where the direct contact with the extracellular matrix (ECM) occurs.

Cells in contact with a stiff surface typically develop discrete multiprotein complexes under the membrane named focal adhesions (FAs), which are the main hub of cell-ECM interaction.

Focal adhesions mechanosensing activity consists in perceiving and transferring the mechanical cues arising from the extracellular milieu to the cellular cytoskeleton. To do so, they are built as complex structures, that can be divided in a transmembrane and in an intracellular layer. The intracellular layer of FAs is composed by scaffolding, docking, and signaling proteins that collectively serve as interface between the transmembrane components directly contacting the ECM (integrins) and the actin cytoskeleton. The molecular composition of FA core is extremely variable and sensitive to ECM composition and mechanics, as perceived by integrin binding. In fact, different degrees of integrin clustering, as determined by the spacing and availability of ECM adhesion sites, affect the recruitment of FA proteins to the binding site (Cavalcanti-Adam et al., 2007; Schiller and Fässler, 2013). Among the proteins composing the intracellular FA layer, some have been shown to be mechano-responsive, while others are mostly known to participate in outside-in signal transduction.

**FIGURE 1:**
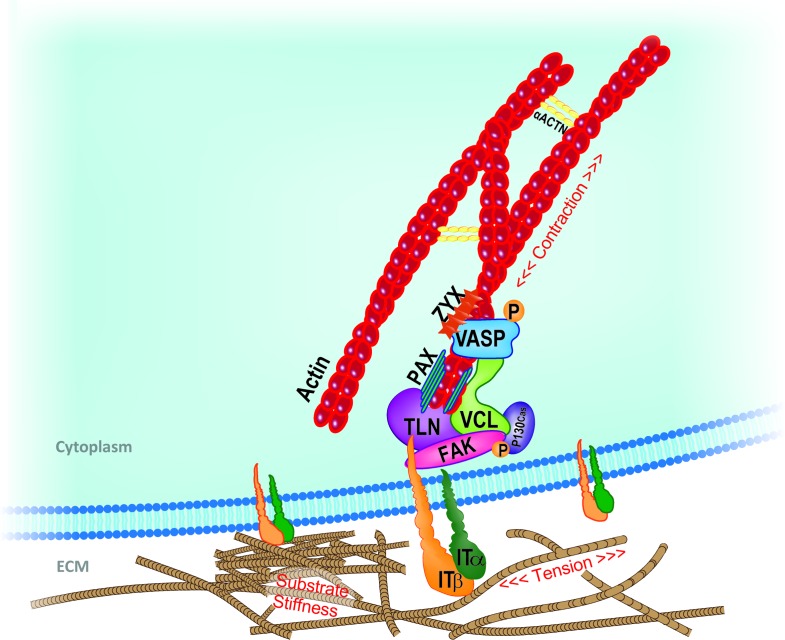
Schematic representation of key mechanosensing players involved in cell-ECM interaction at the focal adhesion (FA) site. Extracellular changes in stiffness, tension or other mechanical stimuli are perceived by integrin clusters whose morphological changes or distribution recruit FAK. Talin rod, vinculin, paxillin, and adaptor protein p130Cas dock to each other and transfer the mechanical cues from integrins to the actin component of the cytoskeleton. In close proximity with the FA inner core, VASP, Zyxin and actinins complex directly regulate actin assembly and dynamics. Adapted from Nardone et al. (2017). ACTN, actinin; FAK, focal adhesion kinase; IT, integrin; PAX, paxillin; TLN, talin; VASP, vasodilator-stimulated phosphoprotein; VCL, vinculin; ZYX, zyxin.

Given the complexity of FA structure and the number and nature of the proteins involved, the modalities by which the FAs act as primary mechanosensor cannot be described collectively; thus, the response of few key FA components to mechanical loading will be herewith described. A representation of the mechanosensing machinery of the FAs is depicted in **Figure 1**.

### Integrin Assembly at Cell-Ecm Interface

The amount of tension generated by a given FA is thought to directly correlate with its size and with the amount of structural, docking and functional proteins recruited to the site (Goffin et al., 2006).

Focal adhesions are dynamically built following the assembly of transmembrane proteins deputed to physically interact with components of ECM, namely fibronectin, vitronectin, collagens, laminins, and named integrins. Integrins are heterodimers composed of α- and β-subunits, whose assembly is guided by ECM composition and whose specificity is given in mammals by the combination of 24 α- and 9 β-subunits and by alternative splicing events. The combination of α- and β-subunits defines the affinity of the receptor for different ECM components and its cell type specificity.

Integrin affinity for its ECM ligand can be regulated within the cell in a process called “inside-out signaling” or prompted by extracellular mechanical stimuli, inducing a high-affinity conformation change (Chen W. et al., 2012). Following such events, integrins are activated, cluster and reinforce molecular links at the cell-matrix interface (Oria et al., 2017; Strohmeyer et al., 2018). Their extracellular domain contacts the ECM, while the cytoplasmic tail interacts with cytoskeletal actin through a number of docking proteins, forming the inner core of the FAs.

Extracellular matrix composition drives precise integrin subsets expression which, being coupled with different signaling cascades, induces specific cellular responses (Seetharaman and Etienne-Manneville, 2018).

Apart from being responsive to changes in ECM biochemical composition and mechanics, cells also adjust their own mechanical state by altering cytoskeletal architecture, modulating cellular elasticity, or generating a concomitant contractile response to applied forces (Webster et al., 2014). The interplay between the external and internal mechanical state of cells is defined by tensional homeostasis, a basal equilibrium stress state in which cells maintain defined levels of tension with their surroundings, despite mechanical perturbations (Brown et al., 1998). In this regard, specific combinations of α-β integrins are known to play different roles in mechanosensing and force generation (Seetharaman and Etienne-Manneville, 2018). Although the mechanical responsiveness of the integrins appears to be diverse, single cell tensional homeostasis is finely tuned mainly by an equilibrium between β3 and β1 integrins (Milloud et al., 2017). In fact, deletion of β3 causes traction forces to increase, whereas the deletion of β1 integrin results in a strong decrease of contractile forces. Interestingly, the distribution of these subunits within the cell membrane is inhomogeneous in static cells, with β1 subunit being more expressed at the perinuclear ring and β3 integrin being restricted to the cell edge (Shiu et al., 2018). Moreover, β3 and β1 integrins have a differential distribution at the leading edge as compared to the rear part of migrating cells (Galbraith et al., 2007) where they trigger distinct signaling pathways (Schiller et al., 2013). Given the incredible number of possible combinations between the subunits and their discrete distribution within the cell membrane, integrins set the pitch of cell mechanosensing at the nanometric scale.

### Focal Adhesion Kinase (FAK)

Focal adhesion kinase is one of the first molecules recruited to developing FA in response to external mechanical stimuli. Its activation by autophosphorylation is considered the trigger to intracellular mechanotransduction, by activating downstream mechanotransducers within the cytoplasm (Lachowski et al., 2018). Downstream signals like cytoskeletal contraction and cell spreading reinforce FAK activation in a positive loop; so FAK phosphorylation can be increased by exogenous force application (such as stretching or resistance by a rigid substrate) (Michael et al., 2009). The interplay between FAK and the contractile cytoskeletal network is tightly controlled in the cell as to maintain tension at critical sites of the cell and to regulate force transfer to the nucleus (Zhou et al., 2015). For example, during processes requiring cell polarization and nucleus deformation, like directional migration, FAK activation occurs at specific sites to favor cytoskeleton local reorganization and nucleus squeezing (Jung et al., 2012). Due to its complexity, the physical mechanism of FAK mechanosensing has been the target of several molecular dynamics and mechano-biochemical network simulations that suggest FAK sensor is homeostatic, spontaneously self-adjusting to reach a state where its range of maximum sensitivity matches the substrate stiffness (Bell and Terentjev, 2017).

### Talin

Talin is a 270 kDa protein composed of an N-terminal globular head, a flexible rod domain and C-terminal helices. While the helices are involved in protein dimerization (Golji and Mofrad, 2014), the head interacts with both β-integrin cytoplasmic domain and F-actin through its FERM domain, recruiting protein 4.1, ezrin, radixin and moesin docking proteins (Ciobanasu et al., 2018). Talin rod features an additional binding site for integrin, and two sites for actin (Gingras et al., 2009). It also contains several binding sites for vinculin, its main partner at the FA site (Gingras et al., 2005). The nanomechanical properties of the protein have been recently characterized and its complexity partly described: talin displays stepwise unfolding dynamics due to the characteristic transition kinetics of its 13 C-terminal mechanosensitive rod subdomains and thus behaves as a force buffer. By stochastic rounds of unfolding/refolding, talin rod domains ensure that force-transmission can be maintained at a low state even across very different talin end-to-end fluctuations (Yao et al., 2016). Altogether, these events set the physiological force range defining the mechanical stability of cell–matrix adhesions (Neumann and Gottschalk, 2016; Yao et al., 2016).

The most recognized effect of force loading to talin consists in its unfolding to expose cryptic hydrophobic binding sites to host vinculin head (del Rio et al., 2009; Hirata et al., 2014; Maki et al., 2017; Rahikainen et al., 2017). In the absence of force, talin rod remains fully structured, and no vinculin binding sites (VBS) are available; under low-force regimes, only the weakest bundle unfolds revealing its VBS. This activates one vinculin molecule, releasing it from its autoinhibited state. As the force applied to talin increases, more bundles are unfolded, revealing more VBSs and thus activating an increasing number of vinculin molecules (Haining et al., 2016). This process is called talin-vinculin mechanosensitivity. In fact, the successful binding of vinculin to talin is considered essential to stabilize talin-F-actin interaction and thus transfer the mechanical signal inward (Humphries et al., 2007).

### Vinculin

Vinculin is one of the main components of FA inner core and its recruitment to the site requires talin activation by mechanical forces (Giannone, 2015): vinculin presence at the FAs correlates directly with the force applied on the same FA (Dumbauld et al., 2013). According to the most recognized model of action, when recruited to the FA, vinculin binds to VBS of talin via its head domain. Once bound to talin at the FA site, vinculin encounters fast conformational changes in its tertiary structure, by switching between an inactive and a low-affinity state (del Rio et al., 2009; Carisey et al., 2013; Hirata et al., 2014). An early model of vinculin mechanosensitivity proposed that the mechanical pertinence of the proteins was conferred by its tail domain. Indeed, cells lacking vinculin show a reduced contractility and this effect can be rescued by transfecting the vinculin tail domain but not the head domain (Mierke et al., 2008). The protein undergoes perpetual cycles of association and dissociation from the FA complex being mediated by its tail domain. Mutants of such domain reinforce FA stability as if the cell was growing on a stiff surface (Rahikainen et al., 2017).

Another model describes a more complex activity: when recruited to FAs, vinculin couples cell area and traction force with differential contribution coming from the head and tail domains. In fact, vinculin transmits force inside-out by increasing ECM-bound integrin–talin complexes via the head domain, while the tail domain is needed to propagate force to the actin cytoskeleton (Dumbauld et al., 2013).

### Paxillin

Paxillin is a 70 kDa phosphotyrosine-containing docking protein being mainly localized at the FA intracellular layer. Here the protein is traditionally believed to integrate mechanical cues arising from the ECM and biological signals propagated via the growth factor receptors. The protein contains different interacting domains (LIM, SH2, SH3 and LD) which confer paxillin high-affinity binding properties to bring together structural and signaling partners (Kadrmas and Beckerle, 2004). The mechanosensing properties of paxillin lie in its ability to bind activated vinculin and paxillin LD motif–binding protein (actopaxin) through the LD domain, thus stabilizing FA-cytoskeleton interaction. To do so, paxillin needs to be phosphorylated by FAK, or by Proto-oncogene tyrosine-protein kinase (Src) on tyrosines 31 and 118. Phosphorylated paxillin exposes additional binding sites for the adaptor molecule Crk, which, in turn, activates the MAPK signaling cascade. The phosphorylation of tyrosine and serine residues in LIM domain has been detected on rigid substrates. Nevertheless, it is still unclear whether these rounds of phosphorylation account for paxillin mechanosensing activity (Bae et al., 2014; Qin et al., 2015).

When extracellular tension is reduced, FA sites lose the ability to recruit paxillin and detach from the relaxed substrate. This event abrogates actin polymerization, resulting in slow actin recovery and increased incidence of stress fiber breaks (Smith et al., 2013). Paxillin has also a shuttling activity which will be described below.

### Zyxin, Ena/VASP, p130^Cas^ and Actinins

Zyxin is a 61 kDa phosphoprotein containing three C-terminal LIM domains and nuclear exclusion sequence (NES). The presence of such domains accounts for its localization to the FAs and for the interaction with a number of FA partners. Zyxin mechanosensing activity consists in its dynamic diffusion through different cell compartments: zyxin is released from FAs when cells are grown on a soft substrate or when the mechanical load is reduced by inhibiting the actomyosin interaction (Uemura et al., 2011). Stretching restores zyxin accumulation in the FAs even in the absence of actomyosin tension, thus demonstrating the mechanosensitive behavior of the protein (Colombelli et al., 2009; Hoffman et al., 2012). Recently, the protein was found to directly regulate F-actin polymerization by interacting with Ena/VASP at the filament barbed end. Zyxin ability to promote actin filament assembly is consistent with its mechanosensitive role in the cytoskeletal reinforcement in response to cyclic stretching (Yoshigi et al., 2005). Zyxin was also found to shuttle to the nucleus. Such activity will be discussed below.

Another zyxin direct interactor is the stretch-sensitive adaptor protein p130^Cas^, recently proposed as a novel mechanosensor (Sawada et al., 2006). P130^Cas^ contains SH3 domains by which it interacts with vinculin and FAK at the FA site. Following integrin clustering and activation, the protein is recruited to the FAs, it unfolds and exposes tyrosine residues that can be phosphorylated. In fact, p130^Cas^ phosphorylation only occurs when cells are stretched (Sawada et al., 2006). The ability of phosphorylated p130^Cas^ to prompt different signaling cascades upon mechanical stimuli, proposes the protein as a hub for the force transmission apparatus with growth factor-stimulated signaling. Additionally, p130Cas-vinculin interaction has been proposed to freeze vinculin in the opened conformation, thus promoting talin binding and FA stability (Janoštiak et al., 2014).

The main role of actinins is in crosslinking F-actin fibers and organizing actin filament cytoskeletal network. The knockdown of α-actinin causes aberrant ECM rigidity sensing, loss of contractility, and enables the cells to proliferate on soft matrices (Meacci et al., 2016). Interestingly, in a compendium of studies, Roca-Cusachs et al. (2013) showed that α-actinin transmits force to nascent FAs, and favor tension-dependent FA maturation. The establishment of this multistep mechanotransduction phenomenon that enables cells to adjust forces on matrices unveil a role of α-actinin that is different from its well-studied function as actin cross-linker (Roca-Cusachs et al., 2013). Furthermore, Lee and Kumar (2016) have determined the mechanical stability and kinetics of human α-actinin-1 highlighting a novel action as molecular shock absorber.

## Cytoskeletal Tension as Second Messenger for Mechanical Signals

The propagation of extracellular and cell-generated forces is ensured by the regulation of cytoskeleton tension (Discher, 2005).

The cytoskeleton is a dynamic structure composed by filamentous and crosslinking proteins. It provides mechanical support to the cells and controls their motility, shape and tension homeostasis (Fletcher and Mullins, 2010). The disruption of cytoskeleton organization can lead to changes in gene expression and the consequent alteration of cell biological response (Tamada et al., 2004; Jaalouk and Lammerding, 2009; Dupont et al., 2011; Iyer et al., 2012).

The mechanical properties of the cytoskeleton depend on the dynamics, geometry and polarity of its components: *actin fibers* (F-actin), *microtubules* (MTs) and *intermediate filaments* (IFs). Each of the components displays a highly organized structure contributing to intracellular organelle integrity and maintenance (Fabry et al., 2001; Chen et al., 2010).

Cytoskeleton contractility is ensured by F-actin sliding on the motor protein myosin II. F-actin and myosin II are held together by crosslinking proteins (e.g., α-actinin, fascin, filamin.) in complex structures called *stress fibers* (SFs).

By pulling on FAs, SFs propagate force from the ECM to the cell and *vice versa* (Cramer et al., 1997; Pellegrin and Mellor, 2007; Naumanen et al., 2008). Based on their structural organization, assembly and FA connectivity, SFs have been grouped in different specialized subtypes (Small et al., 1998; Hotulainen and Lappalainen, 2006). Anchored to FAs only at one end, the dorsal SFs do not contain myosin II, therefore only act as stabilizers that cannot contract (Tojkander et al., 2012). Transverse arcs are, instead, curved contractile SFs characterized by a periodic pattern of α-actinin and myosin II and are only indirectly connected to FAs through dorsal SFs. Dorsal SFs and transverse arcs, generated by *de novo* polymerization, directly interact among them by creating a dynamical network from which ventral SFs can be formed (Hotulainen and Lappalainen, 2006). Ventral SFs are contractile acto-myosin bundles rich of myosin II motors, anchored to FAs at both ends and positioned at the cell base. A recently identified subtype of actin fiber with peculiar function is the perinuclear actin cap, composed of actomyosin bundles wrapped around the nucleus and connecting the nuclear envelope to FAs (Khatau et al., 2009). Through this direct connection, mechanical forces propagate directly from cell periphery to the nucleus (Kim et al., 2012; Li et al., 2014; Shiu et al., 2018).

During the mechanotransduction process, SFs and FAs cooperate and stabilize each other. For example, the relocation of FA protein zyxin and other crosslinkers upon mechanical loading fosters SFs reinforcement and increases cytoskeletal tension (Yoshigi et al., 2005; Colombelli et al., 2009; Fabry et al., 2011). On the other hand, SFs contractility prompts vinculin recruitment to the FAs (Yamashita et al., 2014), where the protein participates in FA composition and organization (Pasapera et al., 2010; Carisey et al., 2013).

Many actin-binding proteins dynamically regulate actin cytoskeleton dynamics in response to intra or extracellular stimuli. Nucleation-promoting factors (Arp2/3, profilin), capping proteins, depolymerizing factors (ADF/cofilin), stabilizing proteins and crosslinkers contribute to control the architecture and the mechanical properties of the network (Pollard and Cooper, 2009; Bugyi and Carlier, 2010; Wiggan et al., 2012).

The main process by which actin cytoskeleton is stabilized by tensile force application consists in the inhibition of the actin severing activity of cofilin (McGough et al., 1997; Hayakawa et al., 2011). When active in the dephosphorylated form, cofilin severs F-actin fibers and exposes the barbed end, at which the protein can be depolymerized (G-actin). This event reduces cell tension. On the contrary, upon mechanical stimulation, cofilin is constantly phosphorylated by LIMK, a kinase activated by Rho/ROCK pathway (Fukata et al., 2001; Hayakawa et al., 2011).

ROCK activation by RhoA also induces myosin II activation by direct phosphorylation of myosin regulatory light chain (MLC) mainly at the Ser-19 residue or by inhibition of MLC phosphatase (MLCP) (Amano et al., 1996; Burridge and Chrzanowska-Wodnicka, 1996). MLC phosphorylation induces actin-myosin interaction and the activation of myosin II ATPase generating contractile force.

**FIGURE 2:**
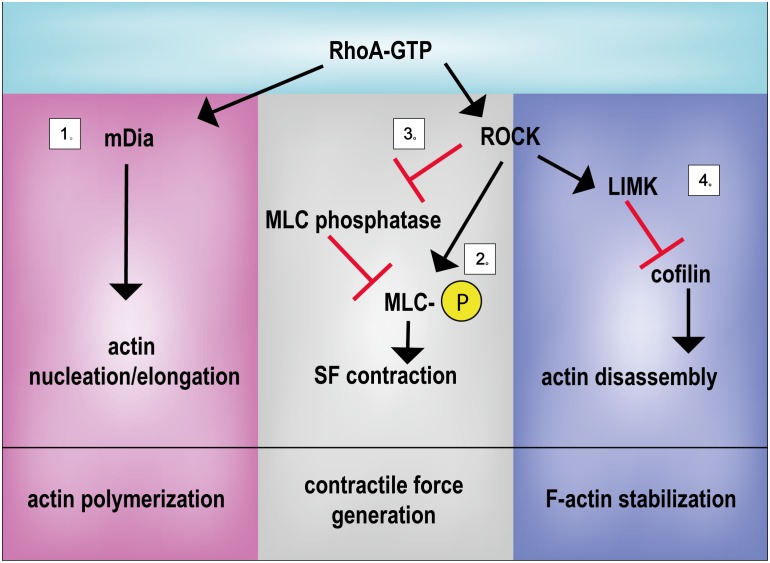
Principal activities of RhoA in controlling mechanical signal propagation. RhoA regulates actin polymerization, contractile force generation and F-actin stabilization by regulating: (1) actin nucleation/elongation through mDia activation, (2) by promoting MLC phosphorylation directly or (3) through MLC phosphatase inhibition and (4) by inhibiting the actin severing activity of cofilin. LIMK, LIM kinase; mDia, Diaphanous-related *formin*-1; MLC, myosin light chain; ROCK, Rho-associated protein kinase; SF, stress fiber.

Besides, ROCK directly participates in cytoskeletal stabilization: Rho/ROCK pathway activates the formin Diaphanous (mDia), which directly or through the Arp2/3 complex promotes F-actin polymerization (Palazzo et al., 2001; Zigmond, 2004; Lessey et al., 2012) (**Figure 2**).

As expected, alterations in the function of Rho or its downstream effectors can affect cell responsiveness to extracellular environment. Indeed, myosin II depletion leads to contractile defects, reduction of FAs, alteration of SFs organization and inhibition of nascent FA maturation (Burridge and Chrzanowska-Wodnicka, 1996; Even-Ram et al., 2007; Cai et al., 2010).

Three isoforms of the motor protein Myosin II (MyoIIA, MyoIIB, MyoIIC) were described in mammals that display different localization, tissue expression and enzymatic properties. As described before, MyoIIA is responsible for generating traction force in order to stabilize FAs in a Rho/ROCK-dependent mechanism. Due to its fast turnover, MyoIIA allows rapid cytoskeleton remodeling. MyoIIB is, instead, an actin fiber stabilizer with no motor function; it is localized at the perinuclear actin cap and involved in maintenance of cell polarity (Kovács et al., 2003).

SFs are physically connected to the MT network (Jiu et al., 2015). MTs, the stiffest cytoskeletal components, are involved in crucial biological processes, such as intracellular trafficking, mitotic spindle formation and cell polarity (Fletcher and Mullins, 2010; Zhang et al., 2014). MTs respond to mechanical stress, as demonstrated by mitotic cells exposed to stretching: following mechanical loading, dividing cells display an alignment of the mitotic spindle parallel to the applied force (Fink et al., 2011). MTs can also affect Rho GTPase signaling via Guanine exchange factor GEF-H1. MTs disruption leads to a higher level of GEF-H1 available for RhoA activation, thus causing SF formation and increased contractility (Krendel et al., 2002). Like MTs, also keratins and vimentin IFs interact with RhoA-GEFs (Solo and GEF-H1, respectively) and control RhoA mediated-SF assembly (Fujiwara et al., 2016; Jiu et al., 2017).

IFs are highly flexible and more stable as compared to F-actin and MTs. Their dynamics and interaction with numerous signaling pathways are regulated by post-translational modifications (Snider and Omary, 2014).

Taking advantage of cytoskeleton-targeting natural compounds or pharmacological drugs (**Table 1**) several groups have identified kinases and transcription factors (Miralles et al., 2003; Olson and Nordheim, 2010; Dupont et al., 2011) modulated by cytoskeletal dynamics.

**Table 1:** Synthetic and natural cytoskeleton targeting compounds.

Category	Target	Compound	Origin	Mechanism	Reference
Actin-targeting compounds	Actin stabilizers	Phallotoxin (Phalloidin)	Natural compound (*Amanita phalloides)*	F-actin binding, ATP hydrolisis and depolymerization inhibition	Estes et al., 1981
		Jasplakinolide	Natural compound (*Jaspis johnstoni*)	F-actin nucleation and polymerization enhancement	Bubb et al., 1994, 2000; Holzinger, 2009
		Cucurbitacin E	Natural compound (*Cucurbitaceae*)	F-actin covalent bound and depolymerization inhibition	Sörensen et al., 2012
	Actin destabilizers	Latrunculins	Natural compound (*Latrunculia magnifica*)	G-actin bound, monomers polymerization prevention	Morton et al., 2000
		Cytochalasins	Natural compound (*Helminthosporium*)	Capping F-actin barbed-ends preventing actin elongation	Brown and Spudich, 1981
		Swinholide A	Natural compound (*Theonella swinhoei*)	Actin polymerization inhibition by G-actin sequestering	Klenchin et al., 2005
		Misakinolide A	Natural compound (*Theonella)*	Inhibition of F actin elongation by sequestering G-actin and capping F-actin barbed ends	Terry et al., 1997
		Mycaloide B	Natural compound (*Mycale izuensis*)	Inhibition of actin polymerization by suppression of actin-activated myosin Mg^2+^-ATPase activity	Saito et al., 1994
Rho/ROCK/Myosin pathway	Rho activators	CN03	Natural compound (Bacterial cytotoxic necrotizing factor)	Deamidation of Gln-63 in RhoGTPase	Flatau et al., 1997
	Rho inhibitor	C. Botulinum C3 exoenzyme	Natural compound *(Clostridium botulinum)*	ADP-ribosylation on Asp41 in the GTPase binding domain	Tautzenberger et al., 2013
	ROCK inhibitors	Y27632	Synthetic compound	Catalytic site competitive binding	Ishizaki et al., 2000
		Fasudil (HA1077)	Synthetic compounds	Catalytic site competitive binding	Yamaguchi et al., 2006
		GSK269962A and SB772077B	Synthetic compounds	Catalytic site competitive binding	Doe et al., 2007
	Myosin II activator	Calyculin A	Natural compound (*Theonellidae*)	Inactivation of phosphatase and promotion of MLC phosphorylation	Peterson et al., 2004
	Myosin II inhibitors	Blebbistatin	Synthetic compound	Block of myosin in an actin-detached state by binding to the myosin-ADP-P_i_ complex	Kovács et al., 2003; Straight et al., 2003
		ML7- ML9	Synthetic compound	Interaction with ATP-binding site of MLCK	Saitoh et al., 1987; Shi et al., 2007
Microtubules	MTs stabilizers	Paclitaxel	Natural compound (*Taxus brevifolia*)	Prevention of MTs disassembly targeting tubulin	Rao et al., 1994; Arnal and Wade, 1995
		Taccalonolides (AF and AJ)	Natural compound (*Tacca chantrieri*)	Regulation of tubulin nucleotide state and GTP hydrolysis inhibition	Wang et al., 2017
	MTs destabilizers	Nocodazole	Synthetic compound	Inhibition of MTs polymerization sequestering free tubulin dimers	Head et al., 1985
		Colchicine	Natural compound (*Colchicum autumnale)*	Prevention of MTs polymerization complexing with tubulin	Skoufias and Wilson, 1992

Actin-targeting compounds are widely used in research to investigate the effect of cytoskeletal integrity and several drugs interfering with cytoskeleton contractility have been recently synthetized. The need for specific inhibitors is a global concern in this field of research: besides perturbing Rho/ROCK pathway and altering cytoskeletal tension, contractility and mechanical properties (Darenfed et al., 2007), the existing drugs may also affect other downstream signaling pathways.

## Mechano-Actuated Shuttling Proteins: Delivering the Message to the Nucleus

The mechanical information arising from modifications of the ECM, perceived by the FAs and propagated at the cytoskeleton level, impacts on proteins residing at the membrane or in the cytoplasm and induces their structural modification and their subsequent shuttling to the nucleus.

Among the first proteins to be identified to shuttle across the nuclear envelope following mechanical signals are the tight junction protein, ZO-1, which accumulates in cell nuclei in a cell density-dependent fashion (Gottardi et al., 1996), tyrosine kinase c-Abl, shuttling from the FAs to the nucleus in response to cell cycle cues (Lewis et al., 1996), and β-catenin, a protein mostly localized at the cell–cell adherens junctions and moving inside the nucleus in response to cytoskeleton remodeling (Gumbiner, 1995; Huber et al., 1996; Orsulic and Peifer, 1996). β-catenin is a component of the cadherin adhesion system at the plasma membrane and has a double function as structural docking protein and as a transcriptional co-activator.

The molecular basis of β-catenin mechanosensitivity have been compellingly demonstrated by single-molecule force spectroscopy (SMFS), showing that the Armadillo Repeat Region (ARM) is mechanically unstable and displays multiple alternative unfolding rounds (Valbuena et al., 2012).

After the discovery of β-catenin shuttling ability, a number of other proteins have been shown to relocate to the nucleus following modifications in ECM composition and mechanics.

Among the proteins sitting at the FAs in static conditions, and shown to detach from the membrane site and move to the nucleus following dynamic stretching, is zyxin (Nix and Beckerle, 1997). As described above, the protein contains a Nuclear Exclusion Signal (NES) that regulates its intracellular localization, and zinc-binding LIM domains, responsible for protein–protein interactions. LIM domains have crucial role in regulating zyxin activity by binding actin at the FA site or transcription factors in the nucleus (Kadrmas and Beckerle, 2004). Although no systematic analysis of its activity as gene expression regulator has been so far provided, a role for zyxin in activating few mechanosensitive genes, like endothelin B receptor (ETB-R), matrix protein tenascin-C and plasminogen activator inhibitor-1 (PAI-1) in smooth muscle cells has been suggested (Cattaruzza et al., 2004).

Paxillin is also credited of having a structural function at the adhesion sites while shuttling to cell nucleus in response to mechanical stress. This protein is predominantly localized to the FAs and its localization can be modified following different rounds of phosphorylation on tyrosine and serine residues by FAK in response to modifications in cell spreading and polarity (Dong et al., 2009; Sathe et al., 2016). Its detachment from the FA complex and its translocation to the nucleus have been shown to be independent of ECM chemical composition, but guided exclusively by mechanical cues (Zhou et al., 2017).

A new class of shuttling proteins acting as mechanotransducers, by moving back and forth from the nucleus without being physically associated to FAs, has been recently described, which will be discussed in detail in the following section.

**FIGURE 3:**
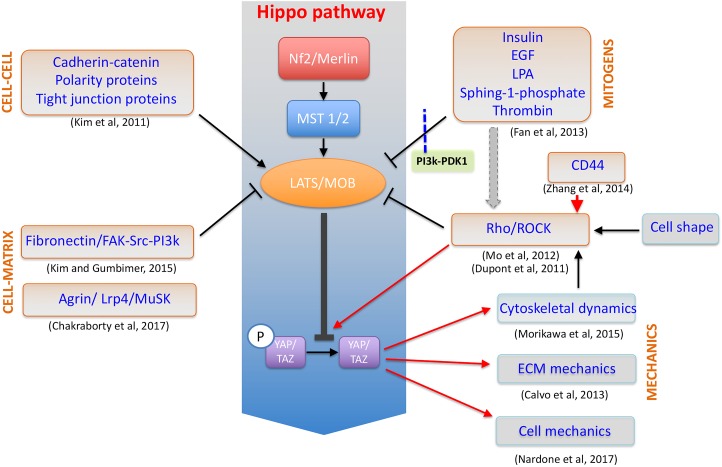
YAP/TAZ at the crossroad of cellular mechanotransduction. Schematic representation of YAP/TAZ factors as the downstream effectors of a number of distinct mechanosensing and biological pathways in the cell and acting to control cytoskeleton dynamics, cell mechanics and in a feed-forward loop to stabilize ECM structure.

Yes-associated protein (YAP) and WW Domain-Containing Transcription Regulator Protein 1 (WWTR1/TAZ) are transcriptional co-activators being the downstream effectors of Hippo pathway (Oka and Sudol, 2009). In response to a number of stimuli coming from the ECM, they shuttle inside the nucleus where they interact with stage- and cell-specific transcription factors to activate a given genetic program. Although being recently credited of exerting rather distinct roles in cell function, the paralog proteins share common structural features (WW, PDZ domains) and are both considered as molecular relays for ECM mechanics given their sensitivity to substrate stiffness (Dupont et al., 2011), cell–cell interaction (Kim et al., 2011) and cell spreading (Nardone et al., 2017) (**Figure 3**).

The definitive demonstration of YAP acting as a mechanosensitive protein was recently given through an elegant experiment performed by the group of Rocha-Cusachs: besides being translocated upon nuclear pore opening following the application of force on the cell, YAP shuttling to the nucleus was shown to depend on the intrinsical protein mechanical instability (Elosegui-Artola et al., 2017).

YAP/TAZ paradigmatic ON/OFF switch-like behavior has been reported in a number of cell types and, if coupled to their acknowledged role in controlling organ shape and size during organogenesis, is the perfect example of how ECM composition and mechanics can impact organ function. YAP/TAZ persistence in the nucleus is regulated by the phosphorylation on specific Serine residues (S127 for YAP, S89 for TAZ) operated by Hippo pathway upstream regulator LATS1/2 and can be released by dynamic modifications in substrate compliance or nanostructure (Mosqueira et al., 2014). Distinct reports have indicated that YAP/TAZ can be sequestered to the adherens junctions by the cadherin-catenin system and by ZO-1 protein (Kim et al., 2011), while a role for Fibronectin/FAK/Src signaling pathway has also been described (Kim and Wirtz, 2015). Evidence that Rho/ROCK-mediated cytoskeleton stability is needed for YAP/TAZ relocation to the nucleus has also been given (Mo et al., 2012), while their sensitivity to mitogens including Epidermal Growth Factor (EGF), Insulin, Thrombin and Lipopolysaccharides (LPA) has been proven (Fan et al., 2013; Haskins et al., 2014). Due to the absence of a nuclear localization sequence in Hippo effectors, the mechanisms involved in their translocation to the nucleus remain elusive for long time. Only recently, the direct association of YAP with the intracellular C-terminal fragment of ErbB-4 has been shown to promote its nuclear localization (Komuro et al., 2003). Moreover, the formation of a shuttling complex YAP/TAZ/SMAD has been shown to be regulated by cell density, with the complex consistently localizing in the nucleus of sparse cells not sensing cell-cell interaction (Grannas et al., 2015).

Since the main annotation for YAP/TAZ transcription targets lies within the proliferation category, the activity of Hippo effectors in the nucleus has been historically associated with cell growth and tumor spreading (Zanconato et al., 2015), while our group and others lately proved that the mechanotransduction role of YAP is to be ascribed to its ability to directly promote the transcription of genes involved in cell-matrix interaction, ECM composition (Nardone et al., 2017) and cytoskeleton integrity (Morikawa et al., 2015). This mechanism has been described as a feed-forward control system by which YAP is controlled by ECM and in turn alters its composition (Calvo et al., 2013).

## Nuclear Mechanotransduction and Mechanically Activated Transcription Factors

Although recent reports suggested mechanical signals influence the expression of mechanosensitive genes, the molecular processes by which mechanical forces are transmitted from the periphery to the nucleus of the cell, the largest and stiffest organelle in eukaryotic cells, are still largely unknown (Dahl et al., 2008). In fact, only lately, few studies suggested the possibility that the nucleus possesses its own mechanosensitive apparatus (Wang et al., 2009; Cho et al., 2017).

The existence of a connection between nucleus and cell membrane has been proven by experiments showing that the application of mechanical forces on integrin receptors at the cell membrane is followed shortly by nuclear structural reorganization and deformation in the direction of the pulling force (Guilak, 1995; Guilak et al., 2000; Jaalouk and Lammerding, 2009; Neelam et al., 2015) and by chromatin reorganization (Booth-Gauthier et al., 2012).

The nuclear-cytoskeletal coupling is crucial for force transmission to the nucleus and, consequently, for the biological response. Many studies have pointed at the nuclear envelope as a regulator of biochemical and physical connection between nucleus and cytoskeleton (Crisp et al., 2006; Fedorchak et al., 2014; Uzer et al., 2016). Indeed, the inner (INM) and the outer nuclear membrane (ONM) of the nuclear envelope host the complex responsible for tying together nucleoskeleton, nuclear envelope and cytoskeleton: the Linker of Nucleoskeleton and Cytoskeleton (LINC).

**FIGURE 4:**
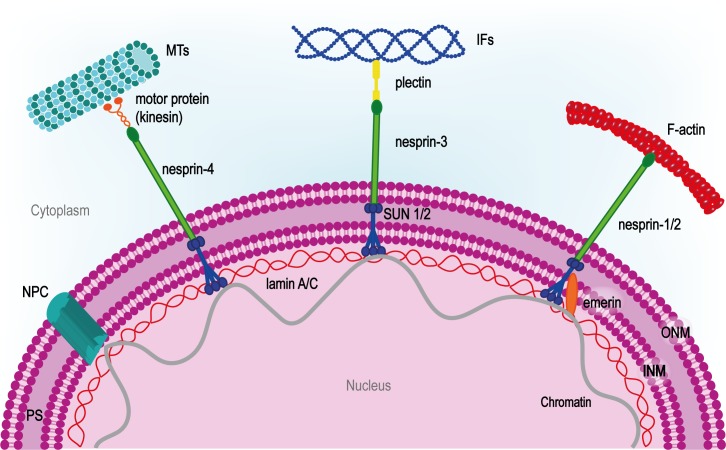
LINC complex at the center of nuclear-cytoskeletal coupling. On the cytoplasmic side, different nesprin isoforms connect the nucleus to the cytoskeleton. Nesprin-1/2 directly bind actin, nesprin-3 is connected to intermediate filaments (IFs) by plectin and nesprin-4 binds microtubules (MTs) through kinesin-1 or other microtubule motor proteins. In the perinuclear space (PS), nesprins bind SUN proteins which span the inner nuclear membrane (INM) and interact with the nuclear lamina through lamin A. The inner nuclear membrane protein emerin anchors SUN protein to lamin A and interacts directly with chromatin. NPC, nuclear pore complex.

The main components of LINC system so far identified are SUN and nesprin proteins. Although different isoforms for each class have been identified, SUN-1/2 and nesprin-1/2 are the most widespread. SUN proteins contain an N-terminal nucleoplasmic region followed by a transmembrane helix at the INM, and the SUN domain at the C-terminal tail. Thanks to this peculiar structure, SUN proteins organize in trimers that span through the INM and bind the C-terminal KASH domain on nesprins in the perinuclear space (Sosa et al., 2012). Nesprins project through the ONM to establish a strong connection between the two nuclear membranes (Crisp et al., 2006). On the cytoplasmic side of the nucleus, multiple nesprin isoforms either bind the cytoskeleton directly or through molecular linkers such as kinesin-1, plectin or dynein (Méjat and Misteli, 2010; Taranum et al., 2012) (**Figure 4**). SUN proteins also interact with nuclear pore complexes (NPC) controlling their organization and distribution on the nuclear envelope (Liu Q. et al., 2007).

LINC components gather at nuclear apical region to form the so-called transmembrane actin-associated nuclear line (TAN) (Luxton et al., 2010). Similar to FAs, TANs appear as discrete spots where LINC proteins get in contact with cytoskeleton and nucleoskeleton, and that accumulate upon mechanical stimulation (Lombardi and Lammerding, 2011; Chambliss et al., 2013).

LINC perturbation has been associated with actin cytoskeleton derangement (Folker et al., 2011; Ho and Lammerding, 2012), nuclear movements and distortion, changes in signal transduction, centrosome positioning and chromatin dynamics (Burke and Roux, 2009; Fridolfsson and Starr, 2010; Gimpel et al., 2017).

On the internal side of the nucleus, SUN proteins link intimately to the nuclear lamina through the main stabilizer of the INM, the intermediate filament lamin A (Haque et al., 2006; Ho and Lammerding, 2012; Gruenbaum and Medalia, 2015). This interaction is credited of propagating the mechanical stimuli from the cytoskeleton to the nucleoskeleton.

Indeed, changes in lamin A protein levels have been detected when tissue rigidity is modified, with soft substrates inducing its phosphorylation and consequent dissociation from the nucleoskeleton (Swift et al., 2013; Buxboim et al., 2014). Lamin A detachment from the nucleoskeleton or its depletion has been associated with the fragility of the nucleus itself and defective nuclear mechanics (Lammerding et al., 2004).

SUN nucleoplasmic domain and lamin A are connected to nuclear chromatin, thus possibly affecting directly gene regulation (Haque et al., 2006; Camozzi et al., 2014). Lamin A-chromatin interaction can be direct or through regulatory proteins like emerin, an integral membrane actin-capping protein promoting nuclear F-actin polymerization (Plessner et al., 2015).

Emerin has been recently described as a sensor of tension upon mechanical stimulation: in isolated nuclei exposed to mechanical stimulation, emerin undergoes phosphorylation by Src kinase, thus leading to lamin A accumulation at the nuclear envelope and nuclear stiffening (Guilluy et al., 2014; Osmanagic-Myers et al., 2015). Cells lacking lamin A, emerin or other LINC components show reduced expression of genes typically targeted downstream of the activation of mechanosensing pathways. For example, nesprin-1 knockdown prevents YAP nuclear shuttling upon mechanical stimulation, proving LINC complex involvement in YAP/TAZ mechanotransduction pathway (Driscoll et al., 2015). Consistent with this hypothesis, mutation in lamin A/C gene (LMNA) causes YAP signaling pathway deregulation (Bertrand et al., 2014).

In a similar fashion, LINC complex and nucleoskeleton components are involved in Wnt pathway regulation; emerin overexpression prevents β-catenin nuclear shuttling and its activity, whereas emerin-depleted cells show substantial accumulation of β-catenin in the nucleus (Markiewicz et al., 2006).

These results appear more interesting when corroborated by the evidence that lamin A and emerin associate with multiple factors involved in transcription regulation, chromatin organization and mRNA processing, thus implying that mechanical cues can impact on mRNA translation through LINC complex (Wilkinson et al., 2003; Dorner et al., 2007).

The association of transcription regulators, LINC complex and nucleoskeleton components at the nuclear periphery has been linked to both the activation and the repression of transcription. Transcriptional activity has been correlated with chromatin rearrangement at the nuclear periphery, in particular to the interaction between euchromatin and NPCs. NPCs are indeed recognized as active transcription sites connected with both cytoskeleton and DNA (Akhtar and Gasser, 2007; Krull et al., 2010; Ibarra and Hetzer, 2015).

Another way by which INM proteins can modulate gene expression in a mechanosensitive fashion is represented by reducing the accessibility of chromatin to transcription regulators. Lamin A binds sites of transcriptionally silent heterochromatin at the INM, while transcriptionally active euchromatin is distributed at the center of the nucleus. According to this model, the nuclear periphery can serve as a resting site for transcription factors, sequestering them and preventing their interaction with target genes (Heessen and Fornerod, 2007).

C-Fos represents a well-described example of transcription factor being sequestered by lamin A/C at the periphery of the nucleus (Ivorra et al., 2006; Scaffidi and Misteli, 2008).

The interaction of nucleoskeleton with transcription factors known to be directly activated by mechanical signals deserves more attention: Mega-karyoblastic leukemia 1 (MKL1, also known as MRTF-A and MAL), member of myocardin family, is a mechanosensitive transcription factor which dissociates from G-actin in the cytoplasm upon mechanical stimulation and activates SRF in the nucleus. Alterations in nucleoskeleton organization affects MKL1 pathway as demonstrated by impaired MKL1 nuclear translocation in lamin A/C depleted cells (Ho et al., 2013).

Also, NF-κB, which is mechanically induced to translocate in the nucleus, suffers defects in the nuclear-cytoskeletal coupling (Lammerding et al., 2004).

## Biological Responses to Cellular Mechanosensing

The interpretation of mechanical cues by the cell is completed by the activation of a given genetic program which induces the cell to adapt to the new conditions.

An example of how cells can respond to mechanical conditioning is given by experiments in which mesenchymal stem cells (MSCs) are grown onto surfaces displaying stiffness gradients. Consistent with the acknowledged ability of the cells to perceive different substrate stiffness, MSCs were shown to migrate toward the stiff area, in a mechanism dubbed durotaxis, which is dependent on cytoskeleton dynamics (Vincent et al., 2013). Since stiffness gradients have been identified in a number of pathological conditions, durotaxis appears to be a general attraction strategy for MSCs to fibrotic areas. An interesting compendium to this study provides evidence that vascular smooth muscle cells undergo durotaxis only in the presence of fibronectin *in vitro*, while laminin seems to restrict their response (Hartman et al., 2016), thus highlighting the substrate-specific nature of the phenomenon.

A number of reports described specific effects of substrate stiffness on cell proliferation, like in endothelial cells (Yeh et al., 2012), airway smooth muscle cells (Shkumatov et al., 2015), and dermal fibroblasts (Razinia et al., 2017). Since all these results were obtained by comparing different stiffness values within the physiological range, it is reasonable to assume that stiffer substrates favor cell cycle. However, contrasting statements can be found in literature (Tan et al., 2014). The variability of conditions and models used in *in vitro* studies can lead to discrepancies and different interpretations of results. In order to have a clear overview, the experimental design must consider the stiffness range specific for each organ/tissue. Depending on the function in the body, softer tissues such as brain (1 kPa) and harder tissues such as bone (1 GPa) can be identified (Handorf et al., 2015).

Increased tissue stiffness has been generally associated with diseased conditions and start to be considered as prognostic factor in cancer progression (Wei and Yang, 2016; Reid et al., 2017). A fibrotic tissue can be 10–100 times stiffer than its healthy counterpart: for example, glaucomatous trabecular meshwork stiffness is 80,8 kPa, while the healthy tissue ranges around 4,0 kPa (Last et al., 2011). A general *consensus* exists that tissue-specific progenitors can be induced to maturation when cultured on substrates resembling the physiological and characteristic stiffness of the tissue they belong to. Indeed, neural stem cells (Saha et al., 2008), pre-osteoblasts (Tse and Engler, 2011), myoblasts (Engler et al., 2004) and adult cardiac progenitors (Forte et al., 2008; Mosqueira et al., 2014) acquire the given phenotype when in contact with matrix displaying a compliance similar to the one they experience *in vivo*. An effect of substrate compliance on the terminal differentiation of embryonic (Bhana et al., 2010) and neonatal (Forte et al., 2012) cardiomyocytes has also been demonstrated. Finally, MSCs have also been shown to be sensitive to substrate mechanics while switching between the osteogenic and the adipogenic lineages. It now appears improbable that MSCs could be induced to become neurogenic, when cultured on substrates mimicking neural stiffness environments (Engler et al., 2006). Neural differentiation is clearly beyond the plasticity of progenitors of the mesodermal lineage.

In living organisms, cells reside in physically confined niches where the surrounding cells and scaffolding ECM present spatially heterogeneous and dynamic mechanical cues (Paul et al., 2017). As such, topography is perceived by cells as a tissue-specific feature. Therefore, engineered materials able to mimic the physiological environment are considered a powerful tool to control cell behavior (Nguyen et al., 2016). The surface topography of a substrate can be defined by parameters like roughness, lateral spacing, height and periodicity (Nguyen et al., 2016).

Cells can distinguish between micro- and nano-scale features as demonstrated by MSCs cultured on gratings of different width. MSCs align and elongate to the grating axis and show smaller and more elongated FAs on nanogratings (250 nm width) as compared to microgratings (10 μm width) or unpatterned surfaces (Yim et al., 2007). The spatial distribution and the alignment of the FAs depends on the periodicity of the grid (Teixeira et al., 2006; Teo et al., 2013).

By controlling nanostructured materials periodicity and spacing, as to match integrin size and spacing through nanodots of 8 nm, it was indeed possible to tune integrin clustering and cell adhesion. By increasing the spacing between the nanodots, integrin clustering was abolished and cell adhesion compromised (Comisar et al., 2012). Similar results were obtained by culturing MSCs on vertically oriented nanotubes, where the reduction of lateral spacing enhanced cell survival, migration and differentiation capacity (Park et al., 2007). The reduction of micropattern height was also shown to affect FA maturation and positioning (Seo et al., 2011).

Finally, a broad spectrum of *in vitro* cell confinement models have been proposed with the aim to reproduce cell constraints by controlling cell shape, area and spreading (Poudel et al., 2012). Cell body confinement on micropatterned surfaces has been shown to control the commitment of stem cells to a specific lineage. As a paradigm, single MSCs constrained on micropatterned surfaces undergo adipogenic lineage specification, while osteoblastic lineage is favored on islands allowing cell spreading (McBeath et al., 2004). Lateral or vertical confinement has been instead used to study directional cell migration, thus showing that MSCs switch from the mesenchymal to the amoeboid migration mode when vertically confined (Liu et al., 2015).

## Evidences for Clinical Relevance of Mechanosensing System

Following the concentric scheme used in the previous sections, we can find evidences of the clinical relevance of the different layers of the mechanotransduction apparatus. Mutations or the aberrant activation of the mechanosensing apparatus as well as pathological responses to mechanical stimuli are, in fact, involved in myopathies, fibrosis, atherosclerosis, and cancer (Jaalouk and Lammerding, 2009). Mutations in mechanosensing, structural, and contractile apparatus have been found to be a source of inherited diseases in tissues exposed to continuous mechanical stress, like striated muscle. Integrins have been described to modulate key effectors of cardiac fibrosis, like angiotensinogen, following sustained pressure overload or mechanical stretch (Graf et al., 2000). In a positive loop, angiotensin II activates integrin αvβ3 in the cardiomyocytes (Kawano et al., 2000).

In cardiac muscle, integrins interplay with the dystrophin-sarcoglycan system to mediate the interaction of the contractile apparatus (sarcomere) with ECM at specialized Z-band sites named costameres. The discrete distribution of the costameres in correspondence of the intercalated disks and Z-bands appears as the most efficient way to ensure the transmission of the forces to the sarcomere; indeed, the derangement of the costameres is a common feature of dilated (DCM) and hypertrophic cardiomyopathy (HCM) (Peter et al., 2011).

Due to its involvement in the stretching activity of the cardiomyocytes *in vivo* (Yutao et al., 2006), in beta-adrenergic stimulation, and in hypertrophic response after hemodynamic load (Li et al., 2012), integrin-β1 inhibition results in heart dilation (Stewart et al., 2014).

Interestingly, inherited mutations in integrins are not common cause of myopathies, but mutations and increased expression of integrin-β1 have been associated with poor prognosis in breast cancer (dos Santos et al., 2012).

The next layer of mechanical signaling within the cell is the link between cellular membrane and cytoskeleton represented by mechanosensors talin, vinculin and its muscle isoform metavinculin (Chorev et al., 2018). Increased expression of talin has been associated with tumor invasiveness and metastatic properties (Sakamoto et al., 2010). The molecular mechanism proposed suggests talin-mediated activation of a pro-survival signaling through integrin, which prevents anoikis and favors cancer growth (Sakamoto and Kyprianou, 2010; Jin et al., 2015).

Given its prominent role as a docking protein at the cell-ECM interaction site, vinculin has been historically suspected of being the main FA switch in cancer progression and invasion. Indeed, vinculin is thought to be crucial in controlling cell anchorage to the ECM. Thus its loss or aberrant expression results in cell migration and, potentially, metastasis spreading (Liu M. et al., 2007).

Its activation by substrate stiffening, such as ECM produced by cancer cells, promotes tumor progression through PI3-kinase activation and basal membrane invasion (Rubashkin et al., 2014; Chang et al., 2017).

Interestingly, the shuttling mechanotransducer YAP has been lately described as one of the key determinants in the positive feedback loop fueling cancer spreading: after being activated in cancer-associated fibroblasts, YAP causes the remodeling of the surrounding ECM and possibly favors tumor spreading (Calvo et al., 2013). The derangement of YAP control has been associated with the growth of a number of tumors, including melanoma, liver, prostate, pancreatic cancer and other neoplastic conditions (Zanconato et al., 2016).

Among YAP upstream control switches, Rho/ROCK pathway has been shown to play a role in leukocytes polarization and migration following their adhesion to the endothelium (Filippi, 2016). The increased activity of Rho/ROCK signaling axis in immune cells has been shown to contribute to early atherosclerotic lesion formation (Mallat et al., 2003), vascular remodeling (Kataoka et al., 2002), and is an independent prognostic marker for survival in cardiovascular outcomes (Kajikawa et al., 2014). The balance between beneficial and deleterious effects in cardiac muscle is more nuanced (Surma et al., 2011). Pharmacological studies indicate that Rho/ROCK axis signaling promotes cardiac hypertrophy, whilst cardiomyocyte-specific conditional expression of low levels of activated RhoA protects from ischemic injury (Xiang et al., 2011). On the other hand, mice suffering from the cardiomyocyte-specific ablation of RhoA have normal hearts and develop compensated hypertrophy before becoming more dilated and less fibrotic in chronic phase (Lauriol et al., 2014). Cardiac fibrosis in response to pressure overload can be inhibited by ROCK inhibition (Phrommintikul et al., 2008), while sustained ROCK-1 activation is responsible for cardiomyocyte apoptosis (Chang et al., 2006).

The following layer of mechanotransduction is the nuclear envelope where the signal sensed from the cytoskeleton is transferred into the nucleus. Mutations in proteins contributing to the nuclear-cytoskeletal coupling lead to altered mechanotransduction signaling and cause a broad range of diseases collectively defined as laminopathies (Capell and Collins, 2006; Worman and Bonne, 2007; Prokocimer et al., 2009).

So far more than 600 mutations in LMNA gene, encoding for lamin A and lamin C via alternative splicing, have been described in humans^1^, the majority of which are missense mutations. LMNA mutations can result in defective lamin A processing, alteration in protein stability, assembly and folding (Wiesel et al., 2008; Bollati et al., 2012).

Lamins are expressed in all tissues but laminopathies have specific targets: tissues exposed to mechanical stress as skeletal or cardiac muscle and bone are the most affected by LMNA mutations. Laminopathies present a wide range of phenotypes and can be grouped according to the affected tissue: neuromuscular disorders [Emery-Dreifuss muscular dystrophy (EDMD), limb-girdle muscular dystrophy], cardiopathies (dilated cardiomyopathy), metabolic diseases (familial partial lipodystrophy) and premature aging disorders (Hutchinson-Gilford progeria syndrome, HGPS) (De Sandre-Giovannoli et al., 2002; Worman and Bonne, 2007; Schreiber and Kennedy, 2013; Brayson and Shanahan, 2017).

Skeletal and cardiac muscular dystrophies are the laminopathies identified most frequently and include limb-girdle muscular dystrophy, autosomal dominant EDMD, and congenital muscular dystrophy (Maggi et al., 2016).

Animal experiments show that lamin A/C knock-out mice develop cardiac and skeletal muscular dystrophy and cells isolated from these mice show defects in the nuclear shape, the distribution of nuclear pore complexes and the mislocalization of nuclear envelope components, such as the inner nuclear membrane protein emerin (Sullivan et al., 1999). Interestingly, mutations in the EDMD gene, encoding emerin, or SYNE1 and SYNE2 genes, encoding nesprins, can also result in skeletal or cardiac dystrophies (X-linked EDMD) (Emery and Dreifuss, 1966; Schreiber and Kennedy, 2013; Meinke et al., 2014).

The molecular basis of the laminopathies are still debated. Due to the role of lamin A as a scaffolding protein of the nucleus, LMNA mutations can result in LINC organization impairment and, consequently, in defects in anchoring the nucleus to the cytoskeleton. Indeed, LMNA-mutated or knock-out cells show defective nuclear-cytoskeletal coupling, deranged nesprin-1 positioning and altered TAN line anchoring. They are thus more susceptible to mechanical stress (Lammerding et al., 2004; Folker et al., 2011; Chen C.Y. et al., 2012; Chen et al., 2014; Zwerger et al., 2013). As expected, these defects in mechanotransduction signaling are more severe in contractile cells (Nikolova-Krstevski et al., 2011; Bertrand et al., 2014), in which nucleoskeleton derangement is usually paralleled by the mislocalization of desmin and connexins (Nikolova et al., 2004).

Together with its role as nuclear scaffolding protein, lamin A also functions as an anchor site for chromatin to the nuclear periphery. As such, it interacts with components of the transcription machinery. Therefore LMNA alterations can prompt chromatin derangement and changes in gene expression. Nuclear envelope defects, heterochromatin displacement from nuclear periphery and nuclear membrane fragility are common features in cells obtained from EDMD patients (Fidziańska and Hausmanowa-Petrusewicz, 2003).

Besides, laminopathies can result from defective lamin A processing (Navarro et al., 2006; Worman et al., 2009). Lamin A protein maturation goes through the production of a lamin A precursor, which is eventually processed via post-translational modifications; LMNA mutations can alter lamin A maturation and can cause its precursor accumulation, like seen in patients affected by HGPS, featuring nuclear morphology alteration and chromatin disorganization (Goldman et al., 2004; Scaffidi and Misteli, 2006).

## Discussion

The consensus over the importance of mechanical signals in shaping cell and tissue function started building with the evidence that cell fate (Engler et al., 2006) and function (Bhana et al., 2010; Yeh et al., 2012; Vincent et al., 2013) can possibly be directed by substrate mechanics.

Modifications in the compliance of ECM are typically associated with the onset and the progression of degenerative diseases (Spinale, 2007) and are now recognized as prognostic tools for the progression of solid tumors (Reid et al., 2017).

Indeed, a simple Pubmed search for “*mechanotransduction*”, a term which applies to all the molecular processes contributing to transform physical cues into a biological response (Jaalouk and Lammerding, 2009), returns a steady increase in results in the last few years.

In the present review, we critically analyzed the recent scientific literature to give a comprehensive compendium of the most important pathways being associated with the complex network of cellular mechanotransduction. Within such pathways, we focused on the integrin-activated axis and highlight the proteins which stand out for their ability to encounter modifications in their structure or function in response to changes in ECM mechanics.

It is worth noting that the definition of cellular mechanosensor chosen in the preparation of the present review applies to all intracellular molecules able to perceive and respond to mechanical loading. Although a consensus is still to be found among the research community on the minimal characteristics a mechanosensor should have in order to be defined as such, the one proposed here appears broad enough as to include different molecular species credited of changing their state or function in response to physical stimuli.

It comprises proteins that unfold to expose cryptic binding sites (del Rio et al., 2009), those that encounter post-translational modifications (Sawada et al., 2006; Dong et al., 2009; Hayakawa et al., 2011; Swift et al., 2013; Guilluy et al., 2014; Qin et al., 2015; Sathe et al., 2016; Lachowski et al., 2018), proteins induced to shuttle (Gumbiner, 1995; Gottardi et al., 1996; Huber et al., 1996; Lewis et al., 1996; Orsulic and Peifer, 1996; Nix and Beckerle, 1997; Dupont et al., 2011), or the ones building novel interactions (Humphries et al., 2007) when subjected to mechanical load.

The argument that the mechanosensor definition should be used only to define molecules, mostly proteins, that change their conformation when exposed to mechanical stress comes from a reductionist approach which cannot be extrapolated to this growing field. Instead, a clearer distinction among proteins perceiving the mechanical signal (mechano-sensor), those transducing the information toward the nucleus (mechano-transducer) and those activating target mechanosensitive genes (mechano-actuator) would be beneficial in drawing the borders of this rather new discipline.

**FIGURE 5:**
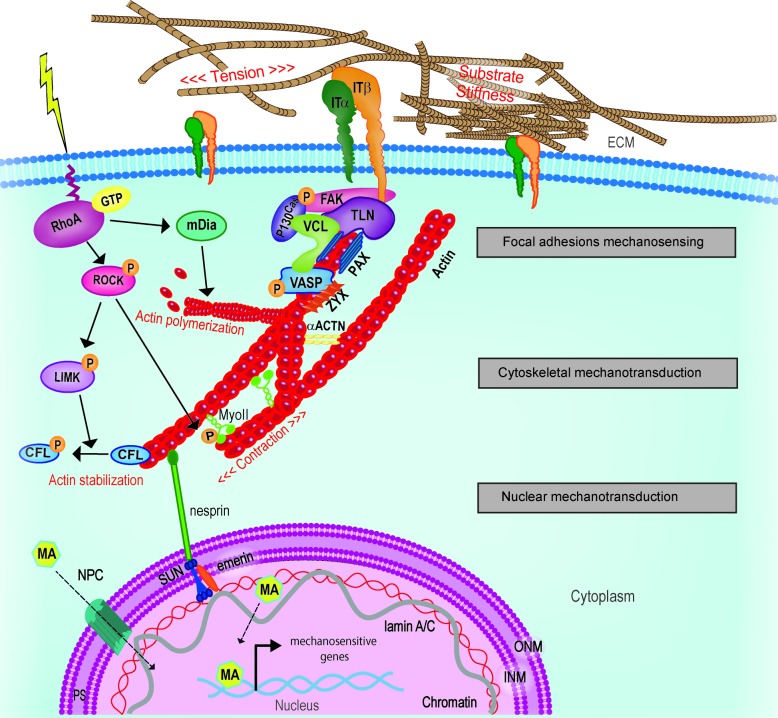
Schematic representation of cellular mechanotransduction layers. Extracellular physical stimuli are perceived by FAs at the cell-ECM interface; the signals are propagate by the cytoskeleton and transferred to the nucleus where mechanosensitive genes are activated by mechanoactuators (MA). MA can be shuttling mechanotransducers or mechanosensitive transcription factors. Adapted from Nardone et al. (2017). ACTN, actinin; CFL, cofilin; FAK, focal adhesion kinase; INM, inner nuclear membrane; IT, integrin; LIMK, LIM kinase; mDia, Diaphanous-related formin-1; MyoII, myosin II; NPC, nuclear pore complex; ONM, outer nuclear membrane; PAX, paxillin; PS, perinuclear space; ROCK, Rho-associated protein kinase; TLN, talin; VASP, vasodilator-stimulated phosphoprotein; ZYX, zyxin.

By adopting these definitions, the intracellular processes favoring the interpretation of mechanical cues can be described in discrete and concentric groups acting to deliver the message coming from ECM dynamic remodeling to the nucleus (**Figure 5**). In this context, the first step is universally recognized as the activation of integrins, which are bound to set the mechanosensing pitch at the nanoscale level (Goffin et al., 2006).

This hypothesis is supported by the evidence that different integrin subsets displaying distinct mechanosensing properties can be expressed in scattered areas of the cell and organized in domains (Shiu et al., 2018). This arrangement suggests that during events like migration, in which the polarization of the cell is required, the cell integrates nanometer-scale mechanosensing response in a timely manner. For this reason FAs rapidly form and break allowing the continuous adjustment and the timely execution of the cellular response (Berginski et al., 2011).

Mechanical signal transduction from the cytoplasm to the nucleus relies on the dynamic regulation of cell cytoskeleton organization and on the tight interplay between specialized contractile structures dispersed in the cytosol and on the nuclear envelope, the latter bridging cytoskeleton and nucleoskeleton (Dahl and Kalinowski, 2011). While the complex regulation of cytoskeleton dynamics is known at least at a certain extent (Discher, 2005), the understanding of the mechanisms by which the tension, propagated through cytoskeleton, regulates the shuttling of mechanotransducers to the nucleus is still elusive. Besides, although recent evidence was provided that force applied on the nucleus can regulate nuclear pores opening and the passive diffusion of mechanotranducers through nuclear envelope (Elosegui-Artola et al., 2017), the processes involved in mechanotransduction at the nucleoskeleton requires further investigation.

**FIGURE 6:**
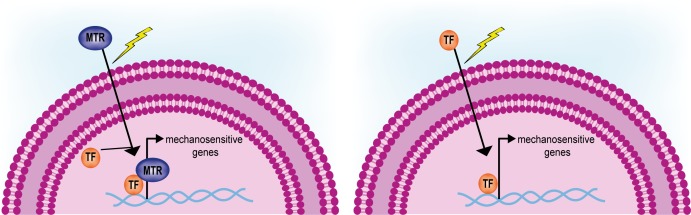
Activation of mechanosensitive genes driven by shuttling mechanotransducers or mechanoresponsive transcription factors. Mechanotransducers (MTR) shuttling from the cytoplasm in response to mechanical stimuli interact and activate given transcription factors (TF, **left**). Only few mechanosensitive transcription factors have been so far identified that are induced to shuttle from the cytoplasm and activate a given genetic program **(right)**.

Additionally, very few studies addressed the modalities of activation of the mechanosensitive genes so far. Two hypotheses can be drawn, which are depicted in **Figure 6**: (1) following the interpretation of the mechanical signals, shuttling mechanotransducers enter the nucleus and function as adaptors for cell- and stage-specific transcription factors; (2) mechanoresponsive transcription factors exist that are only activated or made available for transcription following alterations of ECM mechanics. Although the former appears more realistic in the light of the example of YAP/TAZ co-transcription activators (Dupont et al., 2011), a systematic approach will be needed to rule out the latter.

An important task for future research will be to elaborate integrated strategies aimed at unraveling the interactions among different mechanobiology pathways, which at the moment appear to be intertwined in a complex web (Hansen et al., 2015).

Finally, a further challenge for the future will be represented by the need to scale up mechanobiology studies as to fit a 3D setting, in order to make them more predictive of the *in vivo* situation. This approach would eventually help building more reliable models of mechanosensing failure and identify pathological conditions due to the derangement of the mechanotransduction apparatus.

## Author Contributions

FM proposed the subject and conceived the general structure of the review. FM, AP, VV, and SP revised the existing literature and contributed to all the sections. GF revised the text and contributed the discussion and conclusion.

## Conflict of Interest Statement

The authors declare that the research was conducted in the absence of any commercial or financial relationships that could be construed as a potential conflict of interest.

## Footnotes

1 http://www.umd.be/LMNA/
